# Reinventing social entrepreneurship leadership in the COVID-19 era: engaging with the new normal

**DOI:** 10.1007/s41959-021-00051-x

**Published:** 2021-07-07

**Authors:** Roopinder Oberoi, Jamie P. Halsall, Michael Snowden

**Affiliations:** 1 Department of Political Science, Kirori Mal College, University of Delhi, New Delhi, India; 2 School of Human and Health Sciences, University of Huddersfield, Queensgate, Huddersfield, UK

**Keywords:** COVID-19, Crisis management, Leadership, Management, Social entrepreneurship

## Abstract

In countries across the world, the COVID-19 global health crisis is one of the biggest challenges humanity has faced in recent times. There have been economic, social, political and cultural challenges in all parts of society. Drastic measures have had to be put in place, with many countries injecting extra investment into the health sector and generating support for people who cannot work due to the lockdown rules that have been implemented. The coronavirus pandemic has forced institutions to rethink how the government state functions. Various institutions, from charities and non-government organizations, to the public/private sectors, are the driving forces in tackling this pandemic. Social entrepreneurship is seen as a shining light to public policymakers in these new times, as social entrepreneurship is considered a greater innovator for solutions. The focus of this paper is to critically explore the importance of social entrepreneurial leadership in this new COVID-19 era. In this paper, the authors argue for a rethinking of the connections between social entrepreneurship and leadership and management.

## Introduction

[W]e can define it as the organizing and catalytic effort responsible for bringing about new economic activity (new goods or services) or the provisions of these products in some innovative way […]. Entrepreneurship requires the melding of ideas and opportunities with resources and overcoming whatever constraints lie between the conception and the successful implementation of a project.

(Young, 1987, p. 168)

As Young (1987) states above, entrepreneurship is energizing economic movement in society at different levels. Commonly known today as social entrepreneurship, the concept has become popular with politicians and policymakers. Social entrepreneurship is seen as a driver that creates innovation in different institutions, whether public, private or third sector. Generally viewed as a start-up, social entrepreneurship has two clear altruistic facets—one being for profit and the other being not-for-profit. In countries such as the United Kingdom (UK) and India, there has been a real impetus in social enterprise in recent years. From a social sciences perspective, there has been an avalanche of academic discourse on the subject of social entrepreneurship/social enterprise (see: Halsall et al., 2020a, b; Oberoi et al., 2019a; Park et al., 2017; Samuel et al., 2018). Fundamentally, social enterprise is perceived as a real innovator for social community impact in public circles. Interestingly, Mazzei and Roy note:

The term social enterprise attempts to capture a variety of organisational and legal forms, with different ownership models and motivations driving their engagement in economic activities. The social enterprise research literature has long been concerned with understanding the relationship between public policy and social enterprise (see, for example, Laville et al., 2006) and therefore the role of social enterprise in contemporary societies. For some, the political interest in, and the case for, social enterprise is premised upon the conviction that traditional Western models of welfare provision are coming to an end (Peredo, 2013), and “that welfare states are unaffordable (Roper & Cheney, 2005), bureaucratic and inefficient, and so unable to meet the social needs of citizens (Dees, 1998). This functionalist and managerial account (Dey & Teasdale, 2013) considers social enterprise inevitable, and public policy as the means through which the Third Sector can be transformed into a more efficient mechanism of addressing social needs.

(2017, p. 2452)

It is this supposition that the authors of this paper are focused on, and the suggestion that social entrepreneurship will play an even more crucial role in the current global health crisis. The paper is divided into four sections. The first section provides an overview of the influence that neoliberalism has had upon institutions and the recent antagonistic challenge the ideological framework has experienced. In the second section, the authors present an analytical summary of the COVID-19 pandemic, and how this global health crisis has forced institutions worldwide to press the reset button. Moving on from this, the third section examines the leadership and management challenges that social entrepreneurship could face in a post- COVID-19 global world; as social entrepreneurship leaders are seen as innovators who develop social change and enhance social networks, here, the authors present a six-stage crisis leadership and management model with reference to the COVID-19 global health crisis. By highlighting the ideas in the two previous sections, the fourth section offers some new thoughts for social entrepreneurship and presents a new social entrepreneur avatar framework. Lastly, the paper concludes the main discussions and recommends new areas for social research in an interdisciplinary context.

## Neoliberal institutions and the backlash

In recent times, Neoliberalism has received heavy scrutiny, and, in some academic quarters, there has been an emphasis on crisis and backlash. Boin et al. note that “Crises are commonly considered important moments in organizational history because they offer opportunities to change organizational processes” (2016, p. 76). Moreover, the COVID-19 pandemic “is the latest blow to the ailing liberal international order, which has faced a series of challenges in the post-war era”, as “Beyond the immediate danger COVID-19 poses to human health, its economic, political, and social reverberations are far-reaching” (Norrlöf, 2020, p. 800). However, our current policy dialogue is focused on the doctrine that draws its source from the theory of *homo economicus*—a person as a completely rational actor who constantly pursues his own self-interest. Since the 1990s, Neoliberalism has become well entrenched in global discourse, processes, and structures; it primarily holds that humans are individualistic, self-interested, calculating materialists, which fuels uninhibited free-market capitalism. In his famous 1992 essay, *The End of History and the Last Man*, Francis Fukuyama boldly envisaged the triumph of liberalism after the end of the cold war; he predicted that Neoliberalism would become a catalyst for globalization and the associated liberalization of the economy. Neoliberalism inherently favours competitive market mechanisms over state-controlled or directed economic incentives, and entrepreneurial spirit over cooperative endeavours:

It has been used to explain a wide range of phenomena – from Augusto Pinochet to Margaret Thatcher and Ronald Reagan, from the Clinton Democrats and the UK’s New Labour to the economic opening in China and the reform of the welfare state in Sweden.

(Rodrik, 2017)

The increase of inequality led to distrust and discontent among the masses who were left out of globalization benefits, which thereby initiated the ascendency of a populist anti-globalization discourse and the rise of New Right movements and conservatism. Moreover, Oberoi and Halsall explain that “The expression ‘Lehman moment’ entered the lexicon as a byword for financial calamity, just like ‘Enron moment’ was representational of corporate unprofessional conduct” (2018, p. 6). More than a decade after the global financial crisis of 2008, the clash about the future of capitalist economic models continues, spurred not only by the let-down that precipitated the financial catastrophe, but also by a deepening awareness that the prevailing neoliberal paradigm under-delivered for a large number of people in many geographical areas for many years.

Many recommend civilizing or humanizing capitalism, and the call for conscious capitalism is rising (Oberoi & Halsall, 2018). Indeed, neoclassical economics invariably reflects the dilemma of addressing fundamental developmental concerns: How is sustainable development for the future established? How can equity and equality be guaranteed and how can current models get citizens out of poverty traps? Are global trade, investment, and tax rules stacked against emerging countries? All these vital questions divide the world into the Global South and the Global North, with antithetical ideological positioning on numerous issues. These critical complexities have found little direction in neoliberal equilibrium economics (Gertz & Kharas, 2019).

It is now characteristic to say that globalization is at a crucial intersection. From the appointment of President Donald Trump to Brexit, from the European hostile response towards migration to the rising trade hurdle across the globe, this epoch in global politics is being called the ‘return of history and rise of ideology’. The high-octane cheerfulness of the “end of history” postulation by Fukuyama in the 1990s has given way to the steady trickle of distrust in the capacity of the world system. Throughout this period, there has been a focus on social, economic, and environmental challenges. The liberal hegemonic structural design shaped by the globally privileged sections is being questioned more enthusiastically, and global multilateral organizations are breaking up under their own weight. The last five years have presented a tumultuous political climate, Trump, and Brexit alone we suggest constitute two of the most extraordinary developments in contemporary politics marking neoliberalism’s political victory.

Consequently, for some time now, the anti-neoliberal expression has been distinguished with the anti-globalization movement and continues to animate civil society activists and non-governmental organizations. Indeed, the attractiveness of neoliberalism, which peaked in the 1990s, is showing signs of waning. There have been some definite historical precedents of the neoliberal backlash led by civil society movements like the Battle at Seattle. However, the current times feel somewhat more unusual, because now the challenge to neoliberal orthodoxy is coming not just from periphery countries, but also from the cathedrals of neoliberalism itself, i.e. the US and the UK. The Washington Consensus is being challenged in Washington itself, and neoliberalism is being interrogated by traditionally conservative global institutions. Reports on growing inequality and unsustainable development models are being explicitly talked about. Within academic circles, unorthodox approaches to economic solutions that are questioning the established economic models are gaining traction. For example, as Oberoi observes: “Concomitantly, the race to the bottom, which has become a recurrent theme in the globalization narrative, is now becoming progressively acknowledged as an actuality of globalization” (2015, p. 65). So, there is an ongoing analysis of the whole theory of globalization, which yields extensive literature on both the causes and effects of globalization. The world is at an inflection point, but, with the threat of COVID-19, this has become part of the lexicon of our everyday lives. In his prophetic November 2019 essay, Joseph E. Stiglitz predicted “The End of Neoliberalism and the Rebirth of History”, which unmistakably emphasizes these solemn apprehensions; he wrote:

today, as we face a retreat from the rules-based, liberal global order, with autocratic rulers and demagogues leading countries that contain well over half the world's population, Fukuyama's idea seems quaint and naïve.

(Stiglitz, 2019)

The globalization prophets had assertively guaranteed to the world that their prophecy was based on the scientific rigor of economic modelling and evidence-based research. Well, after four decades, the reality looks very different: the growth has slowed down, and the benefits of economic growth went intensely to the highest echelons; wages have stagnated, and even when the stock market rapidly raised, income and wealth surged increased, rather than descending (Stiglitz, 2019).

At this instant, as nation after nation quarantines itself and erects borders to deal with the virus, the quintessence of Neoliberalism based on the free flow of ideas, people and goods seem to have surged. The pandemic is challenging the ethos and edifice of the global liberal order, and the vulnerabilities of millions are out in the open. Both coordinated and uncoordinated actions to cope with the COVID-19 outbreak put economic freedoms at risk as a result of declining economic activity and the potential for ‘economic security’ policies consistent with economic nationalism. There is also the danger that the post-COVID-19 world might be driven to the precipice, further entrenching the power of the few. At another level, power-hungry and authoritarian regimes are attempting to capitalize on the pandemic to lock “down on individual liberties and move their countries swiftly toward” dictatorship and constraints (Lent, 2020). COVID-19 has greatly impacted economies across the world, with many countries’ economies affected by unemployment, health disasters, and a drop in the movement of people from one country to another. The COVID-19 pandemic has caused much disruption and calls for an urgent reset of economic, social and political societal structures. Moreover, as Norrlöf notes:

To mitigate the hazardous long-term effects of Covid-19 on liberal international order, governments should renew their commitment to core liberal principles, reducing social and economic inequities including access to quality healthcare. The spectre of broader economic security policies cannot be ruled out if international threats to global supply chains and economic welfare continue to mount, either through a prolonged second wave or long-term effects of the first wave.

(2020, p. 804)

At the systemic level, the most coveted liberal order is suffering the effects of COVID-19; if the crisis deepens and becomes more protracted, this could result in a major paradigm shift. COVID-19 reignites longstanding interrogations regarding the agility of the liberal international order in the face of global shockwaves, and predictions for the liberal international order to endure in a world adrift. The effects of COVID-19 have been projected as theoretically momentous and permanent. Other analysts note that some alterations in global affairs are already happening with implications for global leadership and the international order (Zhao et al., 2020).

In a recent article, Oberoi (2020) questions:

1. Are these post-COVID-19 developments manifestations of a beleaguered global capitalist order?
2. Are these shifting concerns in the global order going to strengthen protectionism and boost inward-looking policy regimes?

If so, will the impulse of globalization change permanently? These are some of the emerging issues that require serious attention (Oberoi & Halsall, 2018). It is possible that the only approach that will preserve our society is a “rebirth of history” (Stiglitz, 2019).

## COVID-19 and the big reset in the global agenda

The impact from COVID-19 has stimulated multiple narratives about a major change to global economic ordering. The outbreak of the COVID-19 pandemic has been compared to the Great Depression in the 1930s and the Second World War. The global pandemic has antagonised the world in many ways. The COVID-19 crisis has exacerbated the complications that have dominated the prevailing U.S.-led world order; in fact, it can be likely be credited as the ‘tipping point’ that has altered the rules-based liberal world order. As López (2020) notes:

The COVID-19 crisis is holding up a mirror to Western countries – making us realise that the perception we have of ourselves might be distorted. The crisis will be a huge test: our effectiveness in managing it could alternately accelerate or slow the de-Westernisation of the world. In any case, it will challenge globalisation and rearrange the world order.

As we enter the post COVID-19 world, we need to reckon with the scale and extent of the virus’s blow. There is an increasing belief that Covid19 pandemic may essentially reconfigure the task of state and market for future. The virus has already called into question various established global dimensions and global institutions. The economic fallout of the pandemic has highlighted the helplessness of the global institutional response system. The let down by the World Health Organization (WHO) in providing early warnings to countries has put it under the microscope. Both advanced economies and BRICS countries (Brazil, Russia, India, China and South Africa) have taken hasty steps to close borders and have shifted to restrictive trade and investment measures, further hastening an already emerging shift (Cotula & Schwartz, 2020).

The Covid-19 pandemic has distorted the lives of the 7.8 billion people in the world, forcefully pushing governments and markets to reassess the socioeconomic structures of the new normal (UNDP, 2020). The alarm triggered by the pandemic has wreaked havoc on global financial markets, and there have been serious warnings about a global recession, which could be much worse than the 2008 financial crisis. COVID-19 will impact the global economy in three significant ways: (1) it will directly affect production, (2) create supply chain and market disruption, and (3) financially impact firms and financial markets (Bachman, 2020). In the US, for example, Reuters reported that since March 2020, approximately 36 million people—which works out to be nearly a quarter of the working-age population—have applied for unemployment benefits (Reuters, 2020). Moreover, the International Monetary Fund (IMF) report reveals that the manufacturing output in many countries has slowed down tremendously, which reflects a sharp fall in demand (IMF, 2020). Entrepreneurs, start-ups, and small businesses have been severely impacted by the pandemic and have been recognized as the most vulnerable to COVID-19’s economic disruptions.

It is estimated that the global economy will grow at − 3% in 2020. This is far worse than the 2008 global financial crisis. Economies such as the US, Japan, the UK, Germany, France, Italy and Spain are projected to shrink this year (Indian Express, 2020). This multifaceted calamity has affected all established organizations and methods functioning globally by directly shifting the improvements made by numerous countries for the Sustainable Development Goals (SDGs), necessitating major alterations to sets of policies and solutions, and severely impacting infrastructure and services. Since the global health crisis began countries across the world have carried out socioeconomic impact assessments. Recent work by the UNDP (2020) notes that countries will experience different economic, demographic, and governance problems, which will impact local communities. High levels of state intervention have helped to alleviate the threat of unemployment for many, safeguarding businesses and those who would be worst affected by their closures. For example, Denmark's proposal is “to pay 75% of the salaries of employees in private companies hit by the effects of the” pandemic and “to keep them and their businesses solvent” (Lent, 2020). Whilst in the UK furlough scheme has been set up to cover 80% of salaries while people who cannot work and in California in the US extra support has been put in place for people who are homeless. This has been achieved by turning hotels into shelters (Lent, 2020).

The Green New Deal is discussed as the foundation program for economic recovery in the USA. The idea of a universal basic income has developed a discussion opinion even for Republican politicians. In India, Finance Minister Nirmala Sitharaman, made known the Atmanirbhar Bharat Abhiyan package to offer support to Medium, Small, and Micro Enterprises (MSMEs) in the form of a raise in credit guarantees to aid the financial recovery of the economy. Developed economies have also carried out immense support packages: “India’s financial stimulus package is 10% of its GDP; Japan's is 21.1%, followed by the US (13%); Sweden (12%); Germany (10.7%); France (9.3%); Spain (7.3%) and Italy (5.7%)” (Indian Express, 2020).

As countries begin to ease their lockdown rules and open economies in a staged way, investments in health, hygiene, personal protective equipment, extensive COVID-19 testing of citizens, and the contact tracing of those infected with COVID-19 are crucial and fundamental steps; but, these actions are proving to be challenging to accomplish, particularly in developing countries. Important components for the re-opening phase contain financial assistance for small and medium business, strengthening public service delivery—especially building infrastructure for e-services, building the resilience of industry, and preparing citizens for the new normal world, which consists of fulfilling all essential prerequisites for safe “co-existence with COVID-19” (UNDP, 2020).

There is broad agreement that COVID-19 has initiated critical rethinking, even among the neoliberal supporters. By default, neoliberalism has no robust answers to the current global crisis. The future world order needs to ensure a more egalitarian distribution of resources. Diverse paths are probable; however, if we are to build a fair and sustainable economic system, citizens can reorganize the basic architecture of the conventional global construct and work towards an egalitarian society. However, this notion is seen to be challenged fundamentally, currently, this is illustrated by a recent report published by ONE (2021) suggesting that richer nations such as the UK, Australia, Japan, and the USA are stockpiling vaccines rather than distributing excess stock to poorer countries, in order to supercharge a truly global response to the pandemic. The implication, by ONE, is that wealthier nations putting their own needs first are acting on nationalist greed. As Samuel Nguiffo puts it, “we will not automatically be rid of our greed, which is what led us here” (Cotula & Schwartz, 2020). This underscores the essential political subject, but we also require ground-breaking officially authorized philosophy to recognize issues and discover ways forward. A new generation of researchers and practitioners who have seen the aftermath of 9/11 terror attack, the global economic disaster, and now COVID-19 are ready to reflect in a different way (Cotula & Schwartz, 2020) be more egalitarian and inclusive.

If we do not invent new ways to make globalization more inclusive, we will face an increase of social confrontations, magnified at the international level. “Hence, global markets are twice as challenging: they are deficient in the institutional directive. This double hazard leaves globalization weak and full of transactional costs and it leaves the pursuit for an ideal globalization a fool’s errand” (Oberoi & Halsall, 2018, p. 23). The COVID-19 pandemic has exposed “the unsustainability of global health development strategies. Its socio-economic impacts feed on pre-pandemic vulnerabilities and inequalities across societies, which must be addressed if countries are to build a more resilient future from the perspective of sustainable and people-centered development” (UNDP, 2020, p. 6). The course of recovery from here on is expected to understand societies and economies increasing and shrinking the waves of the global health pandemic. In this volatile, uncertain, complex and ambiguous (VUCA) world of co-existing with COVID-19, countries and their leadership will want to genuinely devote new capability and competence to swiftly adjust, predict, transform, and cope with risks, and “implement solutions” to build an improved new society (UNDP, 2020, p. 15).

## Leadership and management of social entrepreneurship in post covid world

In any organization, whether public, private, or third sector, leadership, and management play a pivotal role. Leadership and management complement each other and can both are essential for the success of an institution. Both concepts have different meanings but go in hand in hand. Leadership in an organization is all about providing a clear direction and developing an inspiring vision. Răducan and Răducan note that leadership is the:

ability to determine the others to participate in a certain way, being a process of orientation of some people by means of communication and convictions and a complex of elements that regards the trust in the people going to the same direction, the mission of the analyzed system, the collective decision and the motivation of human resources.

(2014, p. 808)

Management, on the other hand, is centered around controlling people who work for an organization. Activities in management include outlining a strategy, line-managing people, and other aspects of human resources. Moreover, Aigbavboa et al. state that management is focused around:

planning, execution and managing the people, resources, and scope of the project. Management within an organization should have the discipline to create clear and attainable objectives; moreover, leadership skills of project managers affect project performance.

(2017, p. 479)

At the centre of the organization are several senior management positions (e.g. Chief Executive, Directors, and Board of Governors). These positions are responsible for running the organization and also shape the organization over time. Senior management demands that an individual is visionary, capable of planning and making tough decisions. It is the making of these tough decisions that can empower an organization to thrive and make an impact on wider society. The recent COVID-19 pandemic has caused organizations to think and plan in different ways (Sheth, 2020). Extra financial pressures have been put on organizations due to the COVID-19 pandemic. In this sense, the crucial point here is how effectively a leader and manager can embrace crisis management. Crisis management has been defined as a concept that “involves temporal (mitigation, preparedness, response, and recovery) and spatial (ecological, social, economic, institutional, and infrastructural) dimensions” (Nohrstedt, 2018, p. 232). Moreover, Nohrstedt (2018) goes on to say that there is a general consensus that crisis management develops relationships, mindfulness of interdependency, trust, and conflict tenacity. The latest research by Al Eid and Arnout (2020) using the work of Barton (2007) notes that there are six key aspects of crisis formulation:

1. Surprise: It means that crises occur without warning, or ring bells, but rather suddenly.
2. Lack of information: This means the lack of information on the cause of this crisis, and the reason is due to the lack of information, especially if it occurs for the first time.
3. Escalation of events: when crises occur, juveniles follow to tighten the noose on decision-makers.
4. Loss of control: all events of the crisis fall outside the ability and expectations of the decision-makers, so they lose control and control.
5. Panic: The crisis causes a state of panic, so the decision-maker will dismiss all those involved in the occurrence of the crisis, or resort to quarrels with his aides.
6. The absence of a rapid, fundamental solution: crises do not give the decision-maker a time or opportunity to reach a careful solution, but rather it is necessary to choose between a limited number of solutions and choose the least harmful.

(2020, p. 2)

Crisis management, therefore, is a complex process for any organization, whether public, private, or third sector. Christensen and Lægreid note that crisis management is vitally important in policy circles as a “major crisis” such as the Coronavirus attacks:

at the core of democracy and governance and hence constitute challenges not only for capacity but also for legitimacy and trust. Planning and preparing for the unexpected and unknown, dealing with uncertainty and ambiguity, tackling urgent issues, and responding to citizens' demands and expectations are crucial and difficult tasks for the public authorities.

(2020, p. 1)

**Fig. 1:**
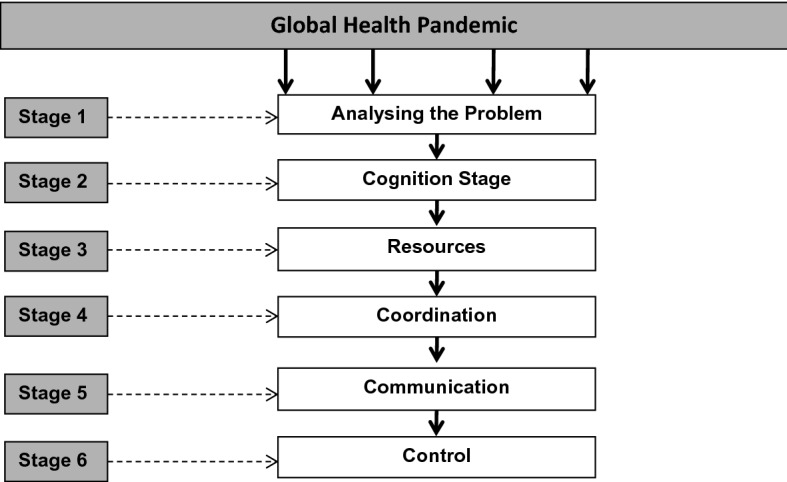
The key stages of leadership and management in a crisis

Here, the COVID-19 pandemic is an example of how senior managers tackle tough issues. When an individual has to engage with crisis management, they are required to consider different facets. In this paper, the authors have developed a flow diagram that explains the key characteristics of crisis management of the COVID-19 pandemic (see Fig. 1). In developing this diagram, the authors have examined the work of Confort et al. (2020), which provides a useful summary of crisis decision-making. As the diagram shows, there are six distinct stages of crisis management that an organization has to go through. Stage one of the process deals with analysing the problem: the senior executives would break up the problem to allow them to obtain a better understanding of the crisis. Stage two is the cognition moment, whereby executives examine the emerging risks to the organization. Stage three deals with resources; this phase is complex as the senior people in the organization must examine what financial resources are needed and what cutbacks are possible. In this type of situation, extra resources are allocated, or re-budgeting occurs to make sure that the key support requirements are being met. Moving onto stage four, coordination involves bringing different activities together (e.g. resources, task/time management) so that the organization can collectively meet its intended goal. In respect of the COVID-19 crisis scenario, Confort et al. note that:

Coping with the risk of COVID-19, each nation faced decisions about how to align the components of their respective national response systems in ways that would slow or stop transmission of the virus, actions that would also contribute to the global goal. Public leaders build trust with their constituents through timely, informed communication, enabling citizens to accept the validity of proposed actions for both self and community and to act, collectively, under the extraordinary constraints of crises.

(2020, p. 617)

Hence, stage five is the communication stage through which the organization conveys its key messages. At present, communication is undertaken via different forms of technology (e.g. email, websites, YouTube, Twitter, Facebook, etc.). The key here is making sure that the message is consistent throughout the different communication means. Finally, the sixth and last stage is control, which means ensuring that the organization responds to outside risks, and crucially, “still maintain[s] regular operations in the society” organization in which the organization works” (Confort et al., 2020, p. 617).

The discussion so far in this section of the paper explains how leadership and management deal with a crisis scenario in a non-specific organization. For a social entrepreneur, running an innovative enterprise will inevitably be more challenging in the current COVID-19 environment. Challenges presented include maintain communication networks with all stakeholders, adapting to changing technology and new ways of working, adapting to public health constraints such as "lockdowns" and service withdrawal, impact upon health and social care. Furthermore, a recent survey by Social Enterprise UK reports that “59% of social enterprises that have responded to our survey so far expect demand for their business to decrease due to COVID-19, with 36% expecting an increase” (2020, p. 7). A social enterprise is reliant upon money coming into the organization; the products need to be bought using the funding that comes into the organization from external agencies and services need to be used by customers. Without these operations sharpening the organization will not exist. In the United Kingdom, it was discovered that:

If social enterprises fail in significant numbers, the potential impact is huge – socially and economically. This is £60 billion of business – or 3% of the economy – which expects to see a 50% decrease in turnover – a potential economic cost of £2.5 billion for each month that the lockdown continues.

(Social Enterprise UK, 2020, p. 7)

If the above projection was born out, this would have a devastating effect on the UK at national and community levels. In particular, social enterprise plays an important role in the health and social care sectors; these types of organizations are at the forefront of communities as they support the most vulnerable groups. Global institutions and the countries’ governments need to ensure that longevity support for vulnerable groups, communities, and societies is provided. This economic longevity would ensure social enterprises would survive the current crisis management scenario and towards an environment of governance normality in the future.

## The new avatar

It is widely accepted that social enterprise and social entrepreneurs are essential and increasingly valuable socially responsible assets in the contemporary global world. Social enterprise and social entrepreneurs influence society by responding to societal challenges, providing solutions to problems presented. Since the pandemic caused by COVID-19, the world has faced fundamental challenges in the way it functions and responds to societal need; a successful response to these challenges requires a critical rethinking of the current strategy, ensuring resources are distributed in a much more egalitarian way. It is reassuring that as Cotula and Schwartz (2020) assert, many are prepared to think differently. Just as the catastrophes associated with 9/11 and the global financial crisis generated new thinking and a renaissance for social enterprise (Oberoi et al., 2019a), so now in the era of COVID-19, social enterprise and social entrepreneurs are presented with further challenges—enabling society to build a new “normal”. Dr. Tedros Adhanom Ghebreyesus, the current Director-General of the World Health Organization (WHO) stated at a briefing in June 2020:

The critical question that all countries will face in the coming months is how to live with this virus. That is the new normal […]. Most people remain susceptible. The virus still has a lot of room to move. We all want this to be over. We all want to get on with our lives. But the hard reality is: this is not even close to being over. Although many countries have made some progress, globally, the pandemic is actually speeding up. We're all in this together, and we're all in this for the long haul. We will need even greater stores of resilience, patience, humility, and generosity in the months ahead.

(WHO, 2020a)

Ghebreyesus reaffirms the views of Executive Director of WHO Health Emergencies Programme, Dr. Michael Ryan (WHO, 2020b) at a COVID-19 Virtual Press conference on the 13th of May 2020, at which the WHO proposed that COVID-19 is becoming endemic within communities. An endemic occurs when there is a constant presence of an infection or disease within a geographic area. For example, Human Immunodeficiency Virus (HIV) is a type of endemic virus, and Dr. Ryan asserts that the global population has a level of awareness about the virus: "HIV has not gone away but we've come to terms with the virus and we've found the therapies and we've found the prevention methods and people don't feel as scared as they did before"; he added that modern medicine is now offering "long, healthy lives to people with HIV" (WHO, 2020b, p. 22). Malaria is another example of an endemic disease in certain geographical parts, such as areas of Africa south of the Sahara, as well as regions like Papua New Guinea. France provides an example of a country that has a mitigation strategy in place to accommodate Malaria and its impact upon the nation. Consequently, drawing upon Ryan (WHO, 2020b) and more recently Phillips (2021) it is clear that Covid 19 is here to stay, and as nations have learned to co-exist with infectious disease, so must countries on a global level learn to co-exist with Covid 19 and develop a new normal.

Clearly, as illustrated earlier in this paper, co-existing with COVID-19 in this volatile, uncertain, complex, and ambiguous (VUCA) world, countries and their leadership will need to devote new capabilities and competence to swiftly adjust, predict, transform, and cope with risks, and implement solutions to build a better new normal. However, the role of the social entrepreneurs in responding to these needs cannot be overstated, indubitably, social enterprise and developing social enterprises are fundamental pillars of the “new normal”. Halsall et al. (2020a) assert that skills associated with the social entrepreneur must be embedded within a graduate's education—ensuring that graduates are able to facilitate and support the development of change in response to social and societal need, both nationally and globally.

Previously, the authors of this paper have asserted that Social Entrepreneurship is “An idea whose time has come”, and that “successful social enterprises are based upon successful social entrepreneurs,” individuals who develop social enterprises are typically altruistic, who want to change things, and “individuals who develop businesses to bring about change” (Oberoi et al., 2019b). Whilst this assertion was made pre-pandemic, it resonates strongly with the challenges presented in the COVID-19 era. As explored earlier in this paper, social enterprise is fundamental to the composition of the social and political economy. The impact and contribution of social enterprises in the growth of societies globally and communities locally should not be disregarded nor underestimated.

Elaborating upon their previous work, the authors of this paper perceive social enterprise to be a complicated, vibrant, and multi-faceted transformationalist practice through which social entrepreneurs offer economic inclusion and social engagement to local communities through imaginative and solution-orientated strategies (Oberoi et al., 2019a, 2020a, b, c). The social entrepreneur is a familiar figure; however, in the COVID-19 era, Snowden et al. (2021) suggest we are presented with a new characterization in the form of a social entrepreneur avatar.

The social entrepreneur avatar proposed by Snowden et al. (2021) is a figure/person who embodies/possesses the characteristics identified by the authors in their current UKIERI-funded project (see British Council, 2019, p. 15). This collaborative venture between Kirori Mal College, the University of Delhi (India), and the University of Huddersfield (UK) has enabled the development of a unique course for students of social enterprise: The Social Innovation and Entrepreneurship Certificate Course provides an avatar of the social entrepreneur for the COVID-19 era. The course focuses upon eight key characteristics of the new social entrepreneur identified by Snowden et al. (2021). Whilst many of the skills and qualities identified reflect those advocated by the British Council (2016), Gunn and Durkin (2016), and Social Enterprise UK (2020), and some do indeed form the bedrock of many courses and workshops delivered to develop entrepreneurial skills. Snowden et al. (2021) propose that bringing these key characteristics together generates a new social entrepreneur avatar for the COVID-19 era.

The social entrepreneur avatar (Snowden et al., 2021) incorporates the development of eight fundamental skills and qualities required to fulfil the role. These include:

### Mentoring

Mentoring asserts (Snowden et al., 2021) is a well-established practice in many professions and has been seen to have mad) e a significant impact upon performance enhancement (Oberoi et al., 2020b), and in the development of organizations. Despite the obvious benefits, mentoring has, as Thomaz and Catalão-Lopes (2019) assert, been largely neglected. Mentoring is described as an “intervention that supports those individuals with less experience within any given context in their personal, social and professional development" (Snowden & Halsall, 2017, p. 297), and an altruistic process that “enables the mentee to access the inside knowledge that the mentor has developed over their life course; distinctly, the mentor is able to translate reality, and help the mentee inhabit their own patterns of reasoning, insight and the application of knowledge and skill” (Snowden, 2019, p. 123).

A successful mentor must be able to facilitate the transfer of knowledge and skills. Whilst it is beyond the remit of this paper to explore these skills in depth, the authors highlight the importance of mentoring skills and practice in a recent Education Committee newsletter (Oberoi et al., 2020a), providing a conceptual framework to illustrate how this process should be constructed and develop. Ultimately, the role of the mentor must be clarified at the outset, using key criteria that underpin a successful mentoring relationship.

### Holism

The term "holism" was first proposed by Jan Smuts in 1926, to describe the tendency in nature to produce wholes from the ordered grouping of substructures or elements, where each part is dependent upon another (Smuts, 1998). However, it is recognised generally that the notion of holism is contextual. For the social entrepreneur, holism means the ability to look at an incident, or setting, as a whole, that is looking at all the sub-components and those facets affecting the issue. A holistic approach will include social, economic, political, psychological sociological, spiritual, physiological, cultural and geographical factors in determining a response. Whilst holism can be viewed as an innate ability, it is something that we all should aspire to (House, 2016). However, to be a successful social entrepreneur, the ability to view matters holistically assert Snowden et al. (2021) is fundamental to success.

### Heutagogy

Heutagogy is the study of self-determined learning and is a process symbiotic with mentoring and holism. Heutagogy is a process of sharing knowledge, but importantly, as Snowden and Halsall (2014) illustrate, it is a process that is learner or practitioner-centric and based upon real-world experiences. Hence, the social entrepreneur has to be grounded in reality, able to make sense of the world they inhabit, to draw upon perceptions and intuition, and demonstrate the ability to conceptualize in a rapidly changing world. A heutagogical social entrepreneur is someone who is able to promote holism, self-worth, and capability, (Snowden et al., 2021).

### Solution focused

Mezirow (2000) defines solution-focused learners as people who look outwards, towards solutions, rather than backwards by reviewing problems. Snowden and Halsall (2014) advise that solution-focused individuals are those with a critical conscience, they are committed, engaged citizens who are familiar with social injustice, oppression, inequality, domination, and sensitive to community needs. Each, are key features and drivers of the social entrepreneur envisaged by Snowden et al. (2021).

### Optimism

Optimism, in the context of the social entrepreneur, relates to an individual with confidence, which in turn creates bold visions when other people are uncertain. Optimistic individuals have a strong sense of self-efficacy and a belief that they have the control necessary to change their circumstances. Seligman (2008) suggests that optimism and an optimistic outlook have the power to enhance outcomes, organization, and performance when presented with challenges; importantly for social entrepreneurs, optimism is a learned behaviour (Seligman, 2004). Therefore, enabling social entrepreneurs to develop coping skills and the ability to think optimistically will strengthen self-efficacy and emotional and social resilience, and in turn, increase the likelihood of success when challenges arise, (Snowden et al., 2021).

### Resilience

Snowden et al. (2021) drawing upon Grant and Kinman describe resilience as “the ability to ‘recover’ from adversity, react appropriately, or ‘bounce back’ when life presents challenges” (2014, p. 24). Littlewood and Holt (2018) comment that there is little research exploring resilience’s relationship with the development of a social entrepreneur; however, they go on to assert and reaffirmed by Snowden et al. (2021) that there is an association between resilience and success. Social entrepreneurs by their very nature encounter challenges and adversity; the ability to overcome these is a desirable quality, and the skills required for resilience, as suggested by Yeager and Dweck (2012), can be learned.

### Empathy

Empathy is described by Snowden et al. (2021) as the ability to place oneself in the position of another person, and imagine perspectives other than one’s own; this is one of the most valuable qualities for a social entrepreneur in terms of understanding the needs of others who they serve. Typically, empathic individuals are those who demonstrate strong emotional and social intelligence, and who are able to connect with others and build strong relationships. However, there is a distinct paucity of research that informs the relationship of empathy with social enterprise and social entrepreneurship. Nonetheless, Bacqa and Altb (2018) and Snowden et al. (2021) suggest that empathy is a crucial attribute that distinguishes social entrepreneurs from customary entrepreneurs, and a significant antecedent of social entrepreneurial motives.

### Creativity

Social entrepreneurs indubitably (Snowden et al., 2021) are the people who bring unconventional ideas to the table, they see new patterns and possibilities that others may not imagine, indeed, Gunn and Durkin (2016) maintain that creativity is an innate skill of the social entrepreneur. However as Haynes (2020) asserts, can be taught and developed as a skill. However, Haynes does acknowledge the importance of context, and for the successful social entrepreneur, creativity needs to be taught in the context of social enterprise.

Society today demands the development of a new social entrepreneur avatar. Humanity is facing a unique challenge, and individuals must be encouraged and supported to apply entrepreneurial approaches to the social problems presented in the era of COVID-19. Oberoi et al. (2019b) allude to this suggesting that “The ingenuity that utilizes entrepreneurial proficiency and spirit to get to the bottom of social problems” whilst not new, is ahead of the conceptual construct, and is crucial to fulfilling the demands of the changing world.

## Conclusion

This paper has been concerned with social entrepreneurship and its key involvement in the COVID-19 global health crisis. The authors firstly focused upon the Neoliberal institution agenda and how this has had to change. As this paper demonstrates, historically, there have been repercussive waves in the backlash of Neoliberalism. The COVID-19 situation has led to scrutiny of the way institutions (i.e. governments) work. This in turn has led to the re-evaluation of the ways in which institutions function in a globalized age and raised questions about the necessity of pressing the reset button on Neoliberalism.

From a leadership and management perspective, the authors have provided an overview of these two concepts and how they have played an important role in social entrepreneurship. Moreover, the paper presented the different critical stages of crisis leadership and management in a post-COVID-19 world. Coupled with this, the authors have drawn upon their previous work to reaffirm the conceptual model of the Social Entrepreneur Avatar, which interlinks with social entrepreneurship, management and leadership.
